# Identifying multimorbidity clusters among Brazilian older adults using network analysis: Findings and perspectives

**DOI:** 10.1371/journal.pone.0271639

**Published:** 2022-07-20

**Authors:** Sandro Rodrigues Batista, Ana Luiza Lima Sousa, Bruno Pereira Nunes, Renato Rodrigues Silva, Paulo César Brandão Veiga Jardim, for the Brazilian Group of Studies on Multimorbidity (GBEM)

**Affiliations:** 1 Department of Internal Medicine, School of Medicine, Federal University of Goias, Goiânia, Goiás, Brazil; 2 Division of Health Care, Goias State Health Department, Goiânia, Goiás, Brazil; 3 School of Nursing, Federal University of Goias, Goiânia, Goiás, Brazil; 4 Department of Nursing in Public Health, Federal University of Pelotas, Pelotas, Rio Grande do Sul, Brazil; 5 Institute of Mathematics and Statistics, Federal University of Goiás, Goiânia, Brazil; All India Institute of Medical Sciences - Bhopal, INDIA

## Abstract

In aging populations, multimorbidity (MM) is a significant challenge for health systems, however there are scarce evidence available in Low- and Middle-Income Countries, particularly in Brazil. A national cross-sectional study was conducted with 11,177 Brazilian older adults to evaluate the occurrence of MM and related clusters in Brazilians aged ≥ 60 years old. MM was assessed by a list of 16 physical and mental morbidities and it was defined considering ≥ 2 morbidities. The frequencies of MM and its associated factors were analyzed. After this initial approach, a network analysis was performed to verify the occurrence of clusters of MM and the network of interactions between coexisting morbidities. The occurrence of MM was 58.6% (95% confidence interval [CI]: 57.0–60.2). Hypertension (50.6%) was the most frequent morbidity and it was present all combinations of morbidities. Network analysis has demonstrated 4 MM clusters: 1) cardiometabolic; 2) respiratory + cancer; 3) musculoskeletal; and 4) a mixed mental illness + other diseases. Depression was the most central morbidity in the model according to nodes’ centrality measures (strength, closeness, and betweenness) followed by heart disease, and low back pain. Similarity in male and female networks was observed with a conformation of four clusters of MM and cancer as an isolated morbidity. The prevalence of MM in the older Brazilians was high, especially in female sex and persons living in the South region of Brazil. Use of network analysis could be an important tool for identifying MM clusters and address the appropriate health care, research, and medical education for older adults in Brazil.

## Introduction

Nowadays, chronic noncommunicable diseases (NCDs) are a major health issue and a challenge to health systems worldwide, including Latin America and Brazil [1–3]. Frequently, individuals with NCDs have more than one chronic disease, and therefore, require complex and individualized care management strategies [4, 5]. The co-occurrence of two or more chronic morbidities in the same person, i.e., multimorbidity (MM) [6, 7]—can occur in all age groups, but predominantly in older adults (64.9% of persons aged 65–84 years and 81.5% of those ≥ 85 years have two or more chronic conditions) [4, 8, 9]. Additionally, MM disproportionately affects socioeconomically disadvantaged populations [10–12].

The impact of a disease in an individual is higher when it is associated with other diseases in contrast to having that disease isolated [13]. MM is a topic of great relevance due to its impact on the negative health outcomes: [14] it is associated to an increase in healthcare spending [15–17], mainly related to medications and potentially inappropriate prescriptions [18] and one of the main determinants of the use of health services [19]. Recent evidence also suggests an important association between the presence of MM and poor health outcomes: poor self-reported health status [20], lower functional capacity [19], lower quality of life [21], higher prevalence of mental disorders [4], and higher mortality rates [22]. Thus, MM’s implications, especially regarding the organization of services and systems of health, have challenged professionals and managers to organize the provision of adequate and good-quality clinical management to these people [4, 23, 24].

In addition to the number of morbidities in a same person, one of the great challenges in the study of MM has been identifying clusters of MM, i.e., which and how many morbidities are more frequently grouped and how they interrelate [25–28]. Attempts to describe possible associative groups of chronic diseases are not recent and include trying to understand which chronic morbidities constitute these patterns, how they develop in the early stages of life, how they are clustered, which morbidities are associated with each other and which are not, and what are the pathophysiological mechanisms that may explain these associations [8, 29, 30].

Clustering is the unsupervised classification of patterns (observations, data items, or feature vectors) into groups (clusters) through a statistical pattern recognition perspective based on similarity. Intuitively, patterns within a valid cluster are more similar to each other than to a pattern belonging to a different cluster [29, 31]. Consequently, cluster identification can support the development of customized and personalized care [31, 32], thereby possibly enabling better health outcomes including reduction of mortality [33, 34]. Researches on chronic disease have used various statistical techniques to analyze this issue: proportion of dyads or triads of diseases, rates of observed/expected prevalence (multimorbidity coefficient), odds ratio, risk ratio, Venn’s diagram, cluster analysis, and factor analysis [27, 35]. Recently, a promising, refined statistical technique called network analysis (NA) has been used to demonstrate the presence of clusters and the complex interactions between coexisting variables by representing the correlation structure of the data [36, 37]. These networks can be used to gain insights into the causal structure of the data, to describe the pattern of predictive relationships in a dataset, or to represent the correlation structure of the data [38].

In this context, studies addressing MM in Brazil are scarce [9, 39, 40], especially studies with a nationwide scope, with an older population and focusing on clusters of MM and the complex interaction between morbidities. Located in South America, Brazil has 209.3 million inhabitants (2017), 84.7% of whom are living in urban areas. In recent decades, there has been a substantial increase in the proportion of older adults in the population and currently 9.2% of the population is aged ≥ 65 years [41]. Thus, in addition to having a rapidly aging population, Brazil has a high prevalence of NCDs, with an increasing disease burden (currently 66%) and significant associated socioeconomic inequalities, forming a hostile scenario for aging [42, 43].

Thus, a better understanding of the epidemiology and complexity of multimorbidity is necessary to address the Brazilian National Health System to delivery an adequate and effective care. Thus, this study aimed: 1) to analyze the occurrence of MM and associated factors in Brazilian older adults and 2) to estimate the MM clusters and the network of interactions between coexisting morbidities.

## Material and methods

### Sample and data collection

This study used data from a nationally representative sample collected during the National Health Survey (abbreviated PNS, from the Portuguese *Pesquisa Nacional de Saúde)*, which was jointly conducted in 2013 by the Brazilian Ministry of Health and the Brazilian Institute of Geography and Statistics (IBGE). A total of 11,177 people aged ≥ 60 years (the legal definition of elderly in Brazil) were interviewed, and they answered a questionnaire with items about their socioeconomic and demographic characteristics and their health status. The sample was devised to be representative of people living in permanent dwellings in both urban and rural areas, covering all 5 geographic regions of the country, its 26 states and the Federal District. A detailed version of the sampling and data collection plan is available in a previous publication [44].

### Variables

#### Multimorbidity

The presence of multimorbidity was considered the outcome variable. To this end, a list of 16 physical and mental morbidities was considered: hypertension, low back pain, hypercholesterolemia, obesity, diabetes, arthritis/rheumatism, depression, heart disease (myocardial infarction, heart failure, or cardiac arrhythmias), cancer, stroke, chronic obstructive pulmonary disease (COPD), asthma/bronchitis, kidney disease, work-related musculoskeletal disorders, other mental health illness, and other chronic diseases. Fifteen morbidities were evaluated just by self-reported medical diagnosis; for each, the following question was asked: “Has your doctor ever told you that you have [each disease from the standardized list]?”. If the answer was yes, the morbidity was counted. In the case of other mental health illness, the question was “Has your doctor or mental health professional (psychiatrist or psychologist) ever told you that you have another mental illness?”. For obesity, anthropometric measurements were used and the participants were classified according to the criteria established by the World Health Organization for the older population [45, 46]. To operationalize multimorbidity, a simple count of morbidities was used, and subsequently individuals have been categorized as no multimorbidity (no MM–no morbidity or one morbidity) or multimorbidity (MM–two or more morbidities) [6, 7, 47].

#### Co-variables

The following co-variables were included in this study: sex (male or female), age (60–69, 70–79, or 80 years and older), skin color (white, black, or brown), marital status (with or without a partner), education (none, up to elementary school, high school, or higher education), having private health insurance (yes or no), geographic area of residence (urban or rural), and geographic region (North/Northeast/Central-West/Southeast/South).

### Statistical analysis

#### Descriptive analysis

The descriptive analyses were done using Stata SE software, version 15.0 (https://www.stata.com), through the *svy* command, which takes sampling weights into account. These weights were defined for the primary sampling units, the households, and all their residents, as well as for the selected inhabitant. Complete information on the sampling weights and the sampling process used in the PNS has been published previously [44]. These results can be extrapolated to the Brazilian population.

Descriptive analysis was used to calculate prevalence (%) and their respective confidence intervals (CI), in addition to the estimated absolute number of people in the population. The prevalence of MM was estimated according to the study’s co-variables. For each of the morbidities, its prevalence in the sample and the average number of other associated morbidities were calculated. Dyads and triads of morbidities were calculated as initial measures of a morbidity cluster.

A multivariate analysis was performed using Poisson regression with backward elimination to consider only variables with possible confounding effects in the analysis. This analysis also took into account the stratification by the presence of MM to evaluate associated factors. For each stratum, all other adjustment variables (sex, age, skin color, marital status, schooling, private plan, area of residence, and geopolitical region) were included in the model at a single hierarchical level. After the first adjustment, the variable with the highest *p* value > 0.20 was identified and excluded from the next adjustment; this process was repeated as long as variables with a *p* value > 0.20 remained in the model. The prevalence ratios (PR) and CIs presented reflect the adjustment for all variables with *p* value < 0.20. Associations with a 95% CI excluding unit 1 were considered statistically significant. The sampling parameters and their weights were considered in all the analyses.

#### Network analysis (NA)

NA was performed to assess the complex relationships between the 16 physical and mental morbidities considered in this study. This refined statistical technique provides the final product of all associations between morbidities, conditional on all associations within the network. Models were constructed for the overall sample to assess the association between morbidities and morbidity networks. These network models comprise and are graphically represented by nodes (circles, representing each morbidity in the model) and edges (lines connecting the nodes). Green edges represent a positive association between morbidities, and red edges represent a negative association. The node size refers to the prevalence of each morbidity for that group [37].

The detection of clusters of morbidities within the network, i.e., the detailing of the network’s overall structure, is performed using a specific algorithm (fast greedy algorithm) that has the ability to detect dimensions of variables efficiently, even when the different dimensions are highly correlated, which makes it superior to factor analyses [48].

The structural importance of each morbidity in the network was analyzed using node centrality measures (strength, closeness, and betweenness) to infer which morbidities were most likely to influence the overall network structure, and which would be the most effective targets for intervention, i.e., those that would have the greatest impact on the model [49]. The direct influence of a given node on the network is measured by its strength. Closeness represents the possibility of information from a given node to “travel,” directly or indirectly, through the network. The power to interrupt the flow of information in the network, characterized by the number of shortest paths in which a particular node is located, defines the measure of betweenness [50]. All analyses related to the morbidity networks were performed using the R Language for Statistical Computing, and more specifically the *qgraph* and *igraph* packages.

### Ethical issues

The Brazilian National Research Ethics Committee approved the PNS on July 8, 2013 under protocol no. 10853812.7.0000.0008. All participants signed an informed consent form before the interviews started.

## Results

### Characteristics of the participants

A total of 11,177 older adults were interviewed, with a mean age of 69.8 years. The majority was female (56.4%). About 53% of the participants declared themselves white and only 9.2% black. Most stated living with a partner (57.4%), one third had no education, and almost a quarter had high-school or higher education (Table 1). The vast majority, proportionally, lived in urban areas and in the Southeast and Northeast regions of the country. Sixty-eight percent reported having no private health insurance.

**Table 1:** Sample description and prevalence of multimorbidity according to the co-variables in Brazilian older adults. National Health Survey (PNS-Brazil, 2013), n = 11,177.

Variables	n	*% weighted*	Multimorbidity	
			%	95%CI
**Sex**				
Male	4,555	43.6	50.7	48.1–53.4
Female	6,622	56.4	64.7	62.8–66.6
**Age** *(in years)*				
60 to 69	6,238	56.4	57.4	55.3–59.5
70 to 79	3,441	30.0	60.5	57.6–63.4
80 or more	1,498	13.6	59.6	55.4–63.7
**Skin color** *				
White	5,314	53.6	60.5	58.3–62.6
Black	1,049	9.2	59.7	54.0–65.1
Brown	4,652	35.6	55.7	53.1–58.2
**Marital status**				
Without partner	6,129	42.6	59.5	57.3–61.7
With partner	5,048	57.4	58.0	55.7–60.2
**Schooling** *(in years)*				
None	3,861	32.1	57.8	55.0–60.4
Until elementary school	4,671	45.6	60.3	57.8–62.7
High school or higher education	2,645	22.3	56.5	53.1–59.8
**Private plan of health**				
No	7,834	68.0	55.8	54.0–57.7
Yes	3,343	32.0	64.5	61.7–67.2
**Geographical area**				
Urban	8,999	85.2	60.8	59.0–62.5
Rural	2,178	14.8	46.3	42.5–50.2
**Region**				
North	1,682	5.4	45.4	41.3–49.6
Northeast	3,394	25.2	53.5	50.7–56.2
Midwest	1,266	6.4	59.5	56.0–63.0
Southeast	3,210	47.9	60.0	57.4–62.6
South	1,625	15.1	67.3	63.6–70.8
**Total**	**11,177**	**100.0**	**58.6**	**57.0–60.2**

* Yellow and indigenous accounted for 1.6% of the sample.

### Prevalence of multimorbidity and associated factors

The occurrence of MM was 58.6% (95% CI: 57.0–60.2) (Table 1). The highest prevalence was observed among women, urban residents, and residents of the Central-West, Southeast, and South regions of Brazil. The occurrence of concomitant physical and mental morbidities occurs in 12.2% (95%CI: 11.2–13.4).

Of the 16 conditions evaluated, hypertension was the most frequent (50.6%), followed by back problems (28.1%), high cholesterol (24.3%), and obesity (23.3%), which affected about a quarter of the sample. The least frequent conditions were work-related musculoskeletal disorders (1.4%) and mental health problems other than depression (0.6%). Other mental illness, kidney disease, and COPD had the highest mean number of associated morbidities: 4.94, 4.65, and 4.39 morbidities, respectively. Individuals with hypertension had the lowest mean number of associated morbidities: 3.12 (S1 Table).

In the adjusted analysis (Table 2), sex and geographic region had a statistically significant association with MM, with women and living in the Southern states having the highest occurrence of multimorbidity. It was not observed association between MM and age group. The skin color was associated only in people with concomitant physical and mental morbidities, being 13.2% (11.8–14.8) in white and 8.5% (6.3–11.4) in participants with black skin. Living with a partner, having private health insurance, and living in urban areas were associated with a higher occurrence of MM (Table 2).

**Table 2:** Adjusted analysis between prevalence of multimorbidity and independent variables in Brazilian older adults. National Health Survey (PNS-Brazil, 2013), n = 11,177.

Variables	Multimorbidity	
	*PR*	*95%CI*
**Sex *(ref*: *male)***		***p<0*.*001***
Female	1.28	1.20–1.36
**Age (in years) *(ref*: *60–69)***		***p = 0*.*379***
70 to 79	1.05	0.98–1.11
80 or more	1.02	0.95–1.10
Skin color* *(ref*: *white)*		***p = 0*.*942***
Black	1.03	0.93–1.13
Brown	1.00	0.94–1.07
**Marital status *(ref*: *without partner)***		***p = 0*.*018***
With partner	1.07	1.01–1.13
**Schooling (in years) *(ref*: *none)***		***p<0*.*001***
Until elementary school	0.96	0.91–1.02
High school or higher education	0.85	0.78–0.92
**Private plan of health *(ref*: *no)***		***p<0*.*001***
yes	1.14	1.07–1.21
**Geographical area *(ref*: *urban)***		***p<0*.*001***
Rural	0.79	0.72–0.86
**Region *(ref*: *North)***		***p<0*.*001***
Northeast	1.17	1.05–1.29
Midwest	1.24	1.11–1.38
Southeast	1.25	1.13–1.38
South	1.42	1.28–1.58

PR: Prevalence ratio. Note: *p-value–The Wald test for heterogeneity*

### Multimorbidity clusters and networks

An initial analysis of morbidity grouping in dyads and triads was performed and the five most frequent dyads and triads of morbidities were presented. Hypertension was present in all combinations, followed by low back high cholesterol (n = 4). The most frequent combination was hypertension and hypercholesterolemia (16.9%), followed by hypertension and low back pain (16.3%) and hypertension and obesity (15.1%) (S2 Table).

**Fig 1:**
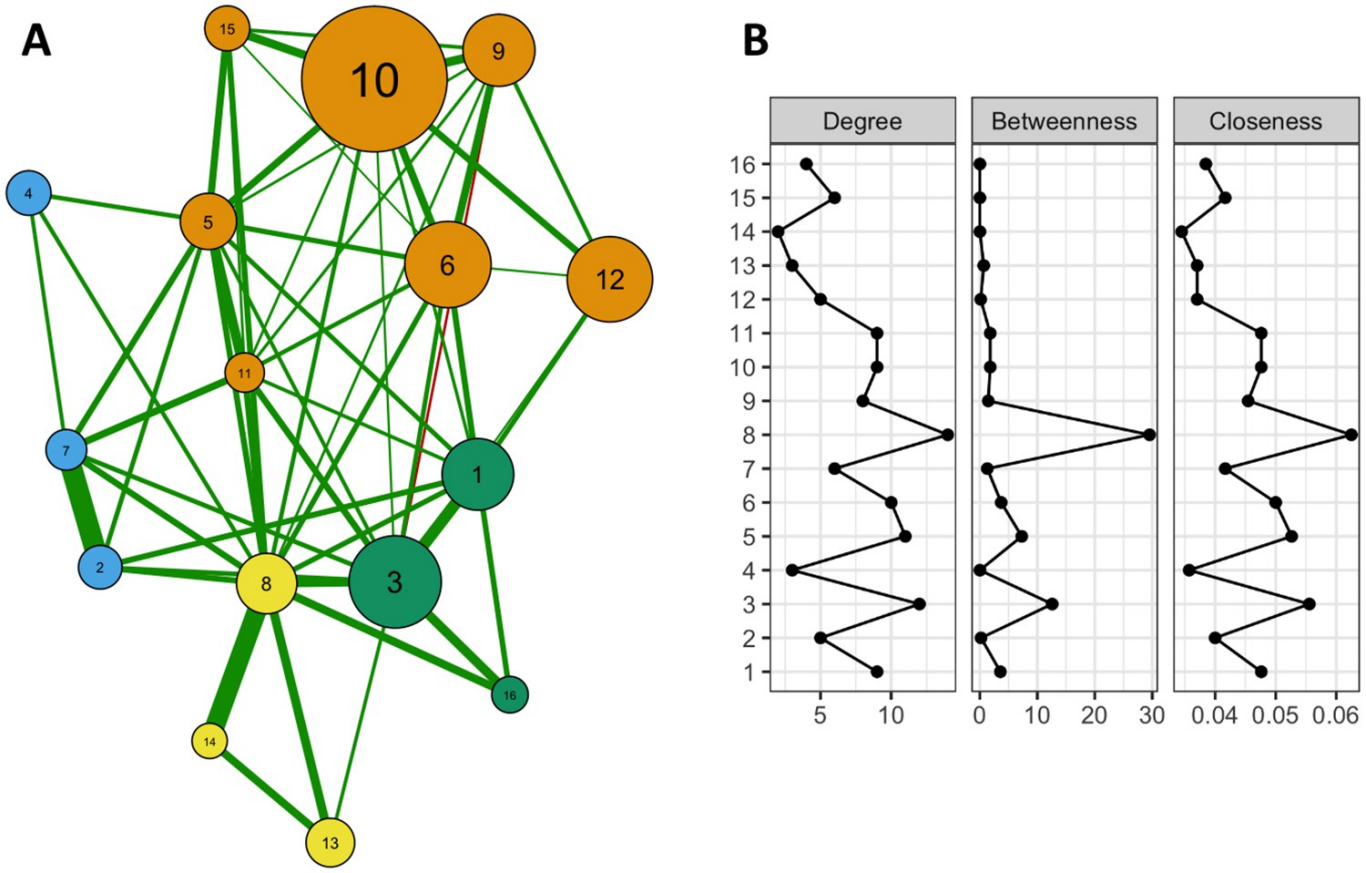
Morbidity network in Brazilian older adults. National Health Survey (PNS-Brazil, 2013). (A) The graph depicts the analysis network. The nodes represent each morbidity in the model, and the edges connecting the nodes represent the effect size for the association between nodes. Green and red edges represent positive and negative connections, respectively. The colors of the nodes correspond to the clusters detected in the network. (B) Network centrality measurements (degree, betweenness, closeness). Morbidities: 1- arthritis/rheumatism; 2- asthma/bronchitis, 3- back pain; 4- cancer; 5- heart disease (myocardial infarction, heart failure, or cardiac arrhythmias), 6- hypercholesterolemia, 7- chronic obstructive pulmonary disease (COPD); 8- depression, 9- diabetes; 10- hypertension; 11- kidney disease; 12- obesity; 13- other chronic diseases; 14- mental illnesses other than depression; 15- stroke; 16- work-related musculoskeletal disorders.

In the general analysis of the morbidity network, four clusters were found in the Brazilian older population (Fig 1A): 1) cardiometabolic, composed of hypertension, hypercholesterolemia, obesity, diabetes, heart disease, stroke, and kidney disease; 2) respiratory + cancer, composed of COPD, asthma, and cancer; 3) musculoskeletal with low back pain, arthritis, rheumatism, and work-related musculoskeletal diseases; and 4) mixed mental illness + other diseases, with depression, other mental illnesses, and other chronic diseases. The morbidity pairs with the highest strength of association were asthma + COPD and depression + other mental illnesses, found respectively in the respiratory + cancer and mental illness clusters. For this general network, the analysis of the centrality measures of the nodes (strength, closeness, and betweenness) showed that depression, heart disease, and low back pain were the morbidities with the greatest influence on the overall structure of the network (Fig 1B). Here, the interpretation of the model suggests that depression is the morbidity with the greatest impact on the model and would probably be the most effective target of intervention.

**Fig 2:**
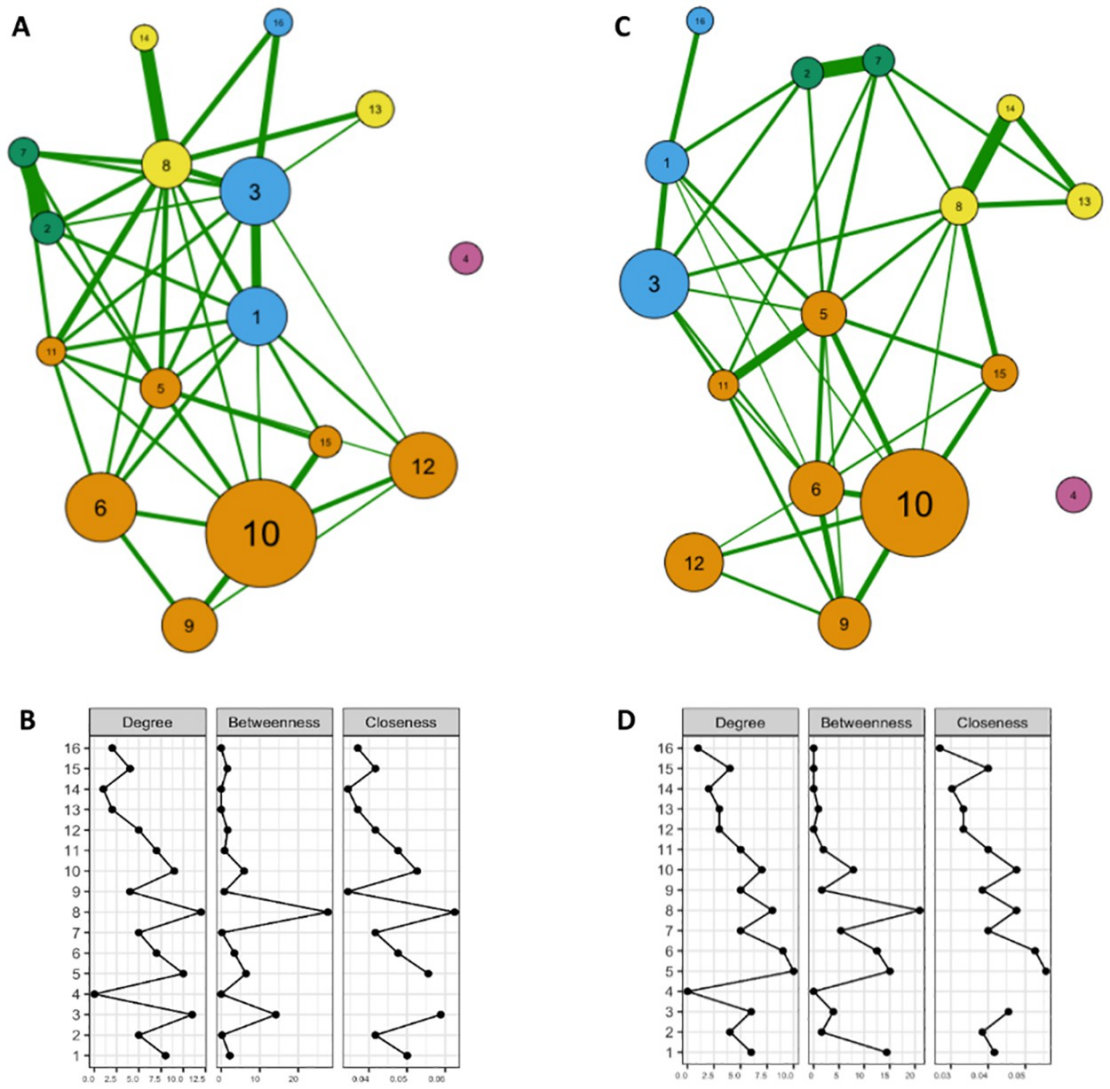
Morbidity network in Brazilian older adults by sex. National Health Survey (PNS-Brazil, 2013). (A) The graph depicts morbidity network for Brazilian older men, n = 4,555. (B) Network centrality measurements (degree, betweenness, closeness) for Brazilian older men. (C) The graph depicts morbidity network for Brazilian older women, n = 6,662. (D) Network centrality measurements (degree, betweenness, closeness) for Brazilian older women. Morbidities: 1- arthritis/rheumatism; 2- asthma/bronchitis, 3- back pain; 4- cancer; 5- heart disease (myocardial infarction, heart failure, or cardiac arrhythmias), 6- hypercholesterolemia, 7- chronic obstructive pulmonary disease (COPD); 8- depression, 9- diabetes; 10- hypertension; 11- kidney disease; 12- obesity; 13- other chronic diseases; 14- mental illnesses other than depression; 15- stroke; 16- work-related musculoskeletal disorders.

When analyzing the network according to sex, it was observed that the network of older men (Fig 2A) started to configure five MM clusters, namely: 1) cardiometabolic, composed of hypertension, hypercholesterolemia, obesity, diabetes, heart disease, and stroke; 2) respiratory, composed of COPD and asthma; 3) musculoskeletal-renal, with low back pain, arthritis, rheumatism, work-related musculoskeletal diseases, and kidney disease; 4) depression, other mental illnesses, and other chronic diseases; and 5) an isolated morbidity (cancer). Kidney disease was no longer in the cardiometabolic cluster and started to be a part of the musculoskeletal-renal cluster. The only cluster that remained unchanged was the mental illness + other chronic diseases. Depression, heart disease, and low back pain, according to the node centrality analyses (Fig 2B), were probably the morbidities with the greatest influence on the overall network structure for older men.

In the analysis of the female-specific morbidity network (Fig 2C), a similarity with the male network was observed, with a conformation of four clusters, plus cancer as an isolated morbidity, except for the non-migration of kidney disease, i.e., this morbidity remained in the cardiometabolic cluster. In the centrality measures of this network’s nodes (Fig 2D), depression (albeit with less intensity), heart disease, and hypercholesterolemia appeared to have the greatest potential to influence the model. Arthritis + rheumatism would become a node with greater influence in the morbidity network of older women.

## Discussion

The present study showed that 58.6% of Brazilian older adults have two or more chronic morbidities. These numbers are alarming: 15,4 million older adults have ≥ 2 diseases and 9,6 million have ≥ 3 diseases. Our study showed the presence of four clusters of MM and the relationship between them and the other morbidities used in the model. According to our knowledge, this is the first study on older adults that evaluates the occurrence of MM and its related clusters with the use of refined statistical techniques to understand the complex relationship between these morbidities in a national cross-sectional study in a LMIC. Older adults with MM are the rule rather than the exception in healthcare systems [5, 17, 51, 52]. Understanding the occurrence of MM and the ways involved on clusters configuration is a complex, prevailing, and an imperative challenge for policy makers and healthcare professionals.

The increased life expectancy associated with improved healthcare systems has allowed the composition of a population group characterized by older adults with multiple chronic conditions [2]. The comparison of multimorbidity prevalence between different countries is a complex task because of a lack of methodological standardization of epidemiological studies [53]. A significant variation occurs in the morbidities considered, in the minimum number of morbidities to be considered, and in the cut-off points used (≥ 2 or ≥ 3 conditions, with or without mental illness) [22]. Even so, in six other middle-income countries [54] the percentage of individuals with MM ranged from 11% (Ghana) to 49% (Russia) in adults aged 60–70 years, and from 16.3 to 66.2% in those aged 70 years or older (nine conditions assessed). In Scotland, half of people aged 50 years have at least one morbidity, and at 65 years, the majority (64.9%) has MM [4]. In some lower-income countries, the prevalence of MM is high and tends to resemble that of higher-income countries [54]. This seems to be the case of Brazil.

In general, the number of morbidities and the proportion of people with MM increase substantially with age and among people living in urban areas [4, 9]. The present study is consistent with those studies. In addition, the highest proportion of older adults with MM was women (64.7%), white (60.5%), and with private health insurance (64.5%), which is consistent with results in the literature [26, 30]. This profile characterizes a group of people with a high use of health services and, consequently, higher diagnosis rates, thereby increasing the reported occurrence of MM [52]. After an adjusted analysis, living in the South Region (one of the most economically developed regions of Brazil) and being female were the variables associated with the highest prevalence of MM.

Our findings point to hypertension as the most frequent morbidity in Brazilian older adults, which is compatible with studies conducted in Brazil and other countries [9, 40, 55]. This higher prevalence of hypertension contributes significantly to the overall prevalence of MM, although this condition has the lowest number of associated morbidities, a finding also reported by other studies [8, 56]. Additionally, despite lower prevalence, people with other mental illnesses, kidney disease, and COPD had more associated illnesses (4.94, 4.65, and 4.39, respectively), similar to the findings in the literature [57].

The understanding of clusters of morbidities and their relationship is imperative for understanding and making decisions about issues related to MM and its outcomes, especially to health systems. In this context, diseases related to the cardiometabolic profile (hypertension, cholesterol disorders, and diabetes) were present in the dyad and triad analyses of the five most prevalent morbidities and also after statistical refinement of the network analysis, constituting a cardiometabolic cluster (hypertension, hypercholesterolemia, obesity, diabetes, heart disease, stroke, and kidney disease). Cardiovascular diseases are some of the most prevalent among individuals with MM [9, 14, 26]. Recent data confirmed these findings among Medicare users and showed that diabetes, anemia, and osteoarthritis were other frequent co-morbidities simultaneously present among people with cardiovascular diseases. The coexistence of cardiovascular diseases in persons that living with MM also indicates a poor prognosis, and the leading cause of mortality among these individuals is heart failure [9, 27, 34, 35].

A systematic review [25] demonstrated the existence of three common clusters of MM (cardiometabolic, mental health problems, and musculoskeletal problems), despite the methodological variability already described among most studies on MM. Other recent studies have also highlighted the high occurrence rate of the cardiometabolic cluster, especially in older adults [8, 35, 55]. The high prevalence of NCDs related to the cardiometabolic system, associated with an increasing disease burden in Brazil, may justify the importance of this cluster in our analysis. Most of the ten leading causes of mortality in Brazil have remained virtually unchanged in recent years. Ischemic heart disease, stroke, and diabetes—all preventable or potentially controllable diseases—continue to account for most of the disease burden among Brazilian older adults [58]. Obesity also plays an important role in the cardiovascular disease burden, and data show that half the Brazilian population is overweight [59].

The cluster related to musculoskeletal diseases is another important group of morbidities, especially in the older population. Low back pain has a high frequency and is one of the leading morbidities in the composition of the disease burden among Brazilian older adults, especially in those aged up to 69 years [58].

The MM cluster that includes more depressive symptoms can be more disabling than combinations that include only somatic conditions [20]. According to the Global Burden of Disease Study Brazil (GBD 2017), depressive disorders are the fourth most important cause of disability, in addition to their impact on the years lived with disability [58, 60].

Among older Brazilians, depression seemed to play a central role in shaping morbidity clusters in older Brazilians of both sexes, since it stood out among the centrality measures in the morbidity network. Depression connects the various clusters, and changes in a given cluster probably only affect other when the depression node also changes. The fact that the depression node is connected to all clusters also explains why it was the node with the greatest closeness measure of all clusters and morbidities in the model. An increasing number of people live with disabilities and limitations arising from depressive disorders in Brazil [60, 61], which demand more mental health resources to increase the availability of prevention programs, early detection, and treatment, in sufficient quantity and quality. Thus, investment in the psychosocial care network, prioritizing the older population, could have a great impact. Finally, these findings support a continued integration between mental and somatic chronic conditions in the conceptualization of multimorbidity, with important implications for clinical practice and health care delivery [20, 51].

The organization of morbidity clusters by sex did not seem to be very relevant, because except for a few morbidities, the respective networks remained very close to the general one. The categorization of clusters by age and sex is still used minimally by researchers, although differences in the epidemiological profile and the clinical practice are known [25, 55, 62]. A study based on records from electronic primary healthcare systems revealed five specific types of clinically consistent patterns of multimorbidity in the adult population: a) cardiometabolic; b) psychiatric + substance abuse; c) mechanical + obesity + thyroid; d) psychiatric + geriatric; and 5) depressive. Two of these (a and c) evolve throughout life and differ in their presentation between the male and female sexes; three of these (1, 3, and 4) affect both sexes; and two are present exclusively in either men or women (2 and 5, respectively). The two patterns that suggested a lifelong evolution were basically composed of risk factors in the younger age group, organic dysfunctions in the middle-aged group, and several disease-related complications in the older group [62].

From a healthcare perspective, the present study also brings important insights for the quality of care in the Brazilian health system. The inadequacy of clinical protocols and evidence-based medicine consolidates a significant and necessary challenge in the care of people with MM [23]. Clinical guidelines that are generally designed for single conditions and exclude “each patient’s unique circumstances” almost entirely disregard individuals with MM. Inappropriate treatment regimens can occur when using guidelines specific to single diseases in individuals with MM, and hazardous effects are likely [63]. The present study reports the main morbidity clusters in the Brazilian older population and thus can support the development of comprehensive clinical protocols based on these associations. Recommendations for these people that living with MM were observed in only 12% and 44% of the clinical guidelines in Australia [64] and the United States [65], respectively.

The present study provides robust information for policy makers and healthcare professionals about the challenges of MM in Brazil. However, some considerations need to be discussed regarding its limitations. First, the use of self-reported information for 15 morbidities may bring up discussions about a possible information bias, specifically related to the memory of the participants or under diagnosis due to barriers in the access to healthcare services among the socioeconomically disadvantaged population. Second, this study used a list of 16 morbidities to characterize MM, and some significant health conditions were not considered, which may reflect in the occurrence rates of the outcome [22]. Moreover, the assessment of MM was based only on a simple count of morbidities, without considering their severity and impact on the individual. Studies that also measure the burden of morbidity are needed, because individuals with the same number of morbidities may require very diverse clinical management and pharmacological treatment [6, 52]. Complementary, another limitation of this study is related to use of obesity and work-related musculoskeletal disorders in our list of morbidities. Despite they have characteristics of chronic conditions individuals with these two conditions don’t have regular care, following or do complementary exams for these. Even that they are conditions which people don’t have a clear self-perception we consider their influence in disease burden and impact in quality of life, especially in older people. Finally, the cross-sectional study design limits the causal interpretation of our findings.

Complementary to this discussion, it is important highlighting the imperative of comprehensiveness approach of elderly care and the role of MM and its related clusters [5, 28, 63]. Other common health problems of old age (cognate decline, dementia, frailty, sarcopenia, falls, for example) have robust relationship to chronic diseases and MM [12, 18, 20, 21, 51, 52]. Thus, there is a complex reality for the individual and health systems involving higher use of health care services (including unnecessary emergence visits), polypharmacy and repeated referrals for specialized care [10, 15, 19, 23]. This study could address the Brazilian health system to improve elderly care for frequent and specific clusters of MM and slow or prevent these related conditions.

In addition of this study, our research team is involved to study the association between this specific clusters and negative outcomes of MM, specially related to clinical situations and health care use. As it is know, clusters of MM could evolve and worsen the quality of life, the burden of disease and treatment. These multifaceted needs must address family doctors, geriatrics, and police markers to develop innovate, effective and resource-efficient long-term care for older people with health-supporting activities each day for optimal wellbeing.

In conclusion, the occurrence of MM and the form of organization and relationship of systematically associated clinical conditions in the older population are important for the decisive issues to redirect both the public and private sectors of the Brazilian healthcare system toward management and assistance centered on individuals and on the improvement of their quality of life, particularly in the case of older adults. Therefore, it is urgent to perform a quantitative and qualitative assessment of the use of healthcare services by older adults with MM and to conduct intervention studies for the development of better standards of clinical practice.

## Supporting information

S1 Table Frequency of morbidities and number of associated morbidities in Brazilian older adults. National Health Survey (PNS-Brazil, 2013), n = 11,177. *Myocardial infarction, heart failure and cardiac arrhythmias.
(PDF)

S2 Table Prevalence of dyads (≥ 10%) and triads (≥ 5%) of morbidities in Brazilian older adults. National Health Survey (PNS-Brazil, 2013), n = 11,177.
(PDF)
