# Supplementary material for: Identifying multimorbidity clusters among Brazilian older adults using network analysis: Findings and perspectives
Source: PLoS One. 2022 Jul 20;17(7):e0271639. doi: 10.1371/journal.pone.0271639 (PMC9299350; doi:10.1371/journal.pone.0271639)
Supplement: S1 Table — National Health Survey (PNS-Brazil, 2013), n = 11,177. *Myocardial infarction, heart failure and cardiac arrhythmias. (PDF) [file pone.0271639.s001.pdf]

S1 Table. Frequency of morbidities and number of associated morbidities in Brazilian older adults. National Health Survey (PNS-Brazil), n = 11,177. Brazil, 2013.

Morbidities	%	Number of diseases *(mean)*
Hypertension	50.6	3.12
Back pain	28.1	3.52
Hypercholesterolemia	24.3	3.70
Obesity	23.3	3.39
Diabetes	18.1	3.53
Arthritis/rheumatism	16.4	3.94
Depression	12.9	4.19
*Cardiac conditions**	11.4	4.03
Other chronic disease	7.1	3.67
Cancer	5.6	3.60
Stroke	4.9	4.07
Chronic Obstructive Pulmonary Disease	3.8	4.39
Asthma/wheezy bronchitis	4.7	4.27
Kidney problem	2.8	4.65
Work-related muscle–skeletal disorders	1.4	4.25
Other mental disease	0.6	4.94

*Myocardial infarction, heart failure and cardiac arrhythmias.
