# Supplementary material for: Identifying multimorbidity clusters among Brazilian older adults using network analysis: Findings and perspectives
Source: PLoS One. 2022 Jul 20;17(7):e0271639. doi: 10.1371/journal.pone.0271639 (PMC9299350; doi:10.1371/journal.pone.0271639)
Supplement: S2 Table — National Health Survey (PNS-Brazil, 2013), n = 11,177. (PDF) [file pone.0271639.s002.pdf]

S2 Table. Prevalence of dyads (≥ 10%) and triads (≥ 5%) of morbidities in Brazilian older adults. National Health Survey (PNS-Brazil), n = 11,177. Brazil, 2013.

**Pairs**	**%**
Hypertension/Hypercholesterolemia	16.9
Hypertension/Back pain	16.3
Hypertension/Obesity	15.1
Hypertension/Diabetes	12.9
Hypertension/Arthritis-rheumatism	10.4
**Triads**	**%**
Hypertension/Back pain/Hypercholesterolemia	6.6
Hypertension/Hypercholesterolemia/Diabetes	6.0
Hypertension/Back pain/Obesity	5.8
Hypertension/Back pain/Arthritis-rheumatism	5.7
Hypertension/Hypercholesterolemia /Obesity	5.6
